# 

**Published:** 1868-01-15

**Authors:** 


*CONTENTS:*

Syphilis. By W. F. Peck, M.D., Davenport, Iowa........... ... 33

Pathological Physiology. The Continous Venous Murmurs in the Neck. Translated by Walter Hay, M.D., Chicago, (Continued from page 13.) 44

Delirium Tremens. By P. S. Macdonald, M. D., Chicago............ 54

Relations existing between the Sense of Touch, etc. By James T. Newman, M.D.............................................. 57

CORRESPONDENCE:

Clinical Cases. By E. R. H.......... ....................... 58

Letter from J. T. Newman, M.D................................ 61

Letter from W. B. Huson, Sheboygan Falls, Wis.................. 61

EDITORIALS:

Ovariotomy............. .................................... 62

LOOT:

Quinine, etc., in Scarlatina...................................... 63

Hypodermic Injection of Quinia in Intermittent Fever, etc......... 63

Bi-sulphate of Lime in Beef Soup, Jellies, etc..................... 64

